# Predictors of relational continuity in primary care: patient, provider and practice factors

**DOI:** 10.1186/1471-2296-14-72

**Published:** 2013-05-31

**Authors:** Elizabeth Kristjansson, William Hogg, Simone Dahrouge, Meltem Tuna, Liesha Mayo-Bruinsma, Goshu Gebremichael

**Affiliations:** 1 School of Psychology, University of Ottawa, Ottawa, Canada; 2 C. T. Lamont Primary Healthcare Research Centre, Bruyère Research Institute, Ottawa, Canada; 3 Department of Family Medicine, University of Ottawa, Ottawa, Canada; 4 ICES UOttawa, Ottawa, Canada; 5 Ottawa Hospital Research Institute, Ottawa, Canada; 6 Trading Regimes Division, Environment Canada, Gatineau, Canada

**Keywords:** Relational continuity, Primary care, Key predictors

## Abstract

**Background:**

Continuity is a fundamental tenet of primary care, and highly valued by patients; it may also improve patient outcomes and lower cost of health care. It is thus important to investigate factors that predict higher continuity. However, to date, little is known about the factors that contribute to continuity. The purpose of this study was to analyse practice, provider and patient predictors of continuity of care in a large sample of primary care practices in Ontario, Canada. Another goal was to assess whether there was a difference in the continuity of care provided by different models of primary care.

**Methods:**

This study is part of the larger a cross-sectional study of 137 primary care practices, their providers and patients. Several performance measures were evaluated; this paper focuses on relational continuity. Four items from the Primary Care Assessment Tool were used to assess relational continuity from the patient’s perspective.

**Results:**

Multilevel modeling revealed several patient factors that predicted continuity. Older patients and those with chronic disease reported higher continuity, while those who lived in rural areas, had higher education, poorer mental health status, no regular provider, and who were employed reported lower continuity. Providers with more years since graduation had higher patient-reported continuity. Several practice factors predicted lower continuity: number of MDs, nurses, opening on weekends, and having 24 hours a week or less on-call. Analyses that compared continuity across models showed that, in general, Health Service Organizations had better continuity than other models, even when adjusting for patient demographics.

**Conclusions:**

Some patients with greater health needs experience greater continuity of care. However, the lower continuity reported by those with mental health issues and those who live in rural areas is concerning. Furthermore, our finding that smaller practices have higher continuity suggests that physicians and policy makers need to consider the fact that ‘bigger is not always necessarily better’.

## Background

Continuity is a fundamental tenet of primary care, and is highly valued by patients; it may also improve patient outcomes and lower cost of health care. Yet, tension exists between continuity and the continuing drive for larger, efficiency driven group practices [1]. In this paper, we explore how the organization of primary care practices relates to an aspect of continuity that is critical to patients [2]: relational continuity.

There are many approaches to conceptualizing continuity of care [3-5]. Continuity may comprise informational [6], relational [3], longitudinal [4,7], team-based [4], management or “continuity coordination” [3], geographical [7] and family continuity [8]. A recent expert working group has delineated three major types: informational, management, and relational [9].

Relational – also variously called personal or inter-personal – continuity refers to an on-going therapeutic relationship between a patient and a provider [3]. Relational continuity refers to a sense of trust and affiliation between patients and their practitioners, often expressed in terms of an implicit contract between them [10]. Patients place a high value on continuity [2,11] and research consistently shows a strong relationship between relational continuity and patient satisfaction [12].

Most empirical evidence on continuity of care indicates that it has a positive effect for patients [13-20] and is associated with lower costs [14,21]. In an extensive review of the empirical continuity literature, Gray and colleagues found strong evidence that relational continuity improved the uptake of preventive care, enhanced adherence to treatment, and increased satisfaction with care [22]. Sans-Corrales and colleagues reported similar findings in a more recent systematic review: relational and longitudinal continuity of care were associated with increased patient satisfaction, improved health outcomes and cost effectiveness in primary care [23]. A critical review revealed that relational continuity had significant positive associations with 51 out of 81 quality of care outcomes; evidence was strongest for relationships between continuity and increased uptake of preventative services [24]. Greater continuity was also significantly associated with 35 of 41 cost of care outcomes, including lower emergency room use, lower hospitalization, and fewer ‘no-shows’ for appointments. Canadian studies have supported a relationship between continuity of care and lower ER use among older adults [25], older men [26] and overall [27]. Continuity was also associated with lower ER use in a longitudinal study in Taiwan [28]. The evidence in favour of continuity is less clear-cut [22] in one area only: chronic disease management.

Despite this evidence, changes in the organisation of primary care and use of information technology in the delivery of health care have challenged relational continuity [1]. In Canada, as in the rest of the world, solo practices are fast dissipating [29,30]. In 2001, only 25% of family physicians in Canada worked in solo practices, down from 31% in 1997 [31]. In the same year, 74% of family physicians worked in group practices, sharing office space, staff, expenses, patient records, and on-call duties. In group practices, especially in larger ones, a decline in relational continuity is likely. It seems that relational continuity of care is losing ground as a principle of health care planning [32]. Given this trend, and the fact that relational continuity contributes to patient satisfaction and better outcomes, it is important to investigate factors that are associated with relational continuity, particularly in a Canadian context. It is also important to understand which models of primary care provide greater continuity. This in turn, can provide important lessons for primary care practitioners and decision makers alike. Yet, to date, little is known about the practice, provider and patient characteristics that predict good continuity of care.

This paper is designed to address these needs. Our purpose was to assess predictors of continuity of care within a large sample of primary care practices in Ontario, Canada. A secondary question assessed whether there was a difference between organizational models of primary care in the continuity of care provided. The present study is part of a larger mixed methods study of primary health care in Ontario: the Comparison of Models of Primary Care in Ontario (COMP-PC).

## Methods

### Design

The COMP-PC project was a cross sectional study of four models of primary care service delivery set in the Canadian province of Ontario between October 2005 and June 2006. The four models comprised Fee-For-Service (FFS) practices (both traditional FFS and reformed Family Health Group models), capitation-based system Health Service Organizations (HSOs), Community Health Centres (CHCs) which employ salaried physicians as well as a multidisciplinary team and focus on community, and a newer model of Family Health Networks (FHNs), which incorporate extended-hour coverage, information technology and a blended remuneration formula of (principally) capitation, performance bonuses and fee for service. Several performance parameters were evaluated, but this paper focuses on a patient reported measure of relational continuity (hereafter referred to as “continuity”). Full details on the methodology for the entire project can be found in a separate publication and are summarized below [33]. The study was approved by the Ottawa Hospital Research Ethics Board.

### Sample

Our practice sampling frame included all known and eligible Family Health Networks (FHN; n = 94), Community Health Centres (CHC; n = 51) and Health Service Organisations (HSO; n = 65) in Ontario. The Fee-for-Service (FFS) sampling frame of 155 represented a random sample extracted from a list of 1,884 practices. We excluded practices that did not offer primary care services for adults, had belonged to their respective model for less than one year and where fewer than 50% of the practices’ providers consented to participate in the survey. Physicians or nurse practitioners were eligible if they had worked at the practice for a year or 6 months, respectively. Patients were eligible if they were over 18 years of age and not cognitively impaired or acutely ill. Informed consent was obtained from all participants.

### Instrument

The Primary Care Assessment Tool (PCAT), a validated tool designed to assess the quality of primary care [34,35], was used as the basis for our work. From this, we adapted a patient survey, a provider survey and a practice survey. The patient survey was divided into two sections. The first part, which included the measure of continuity, was completed in the waiting room before the visit with the provider while the second was completed after the appointment and captured visit-specific information, such as waiting times. Surveys were available in French and English, and translators were used to assist individuals not literate in either language. The provider survey was completed by family physicians and nurse practitioners; it included demographic information on the providers and their perception of practice performance. A single practice survey was completed by the practice manager or lead physician; it contained items describing practice environment (including the team structure, hours of operation, and the availability of medical and social services in the local community).

### Outcome (continuity) measure

A four-item scale included in the patient survey measured relational continuity. Questions covered the extent to which: the patient is seen by the same provider each time, the patient can call and talk to the provider who knows them best, the provider sees the patient as a person, and the providers’ knowledge of which problems are most important to the patient. A proportion score (ranging from .25 to 1.0 with higher scores reflecting greater perceived continuity) was derived from the scale by dividing the score by the maximum possible score of 16. The internal consistency of the scale was 0.68, which indicates moderate reliability.

### Analysis

#### Descriptive and bivariate analyses

Descriptive patient and practice profiles across models were compiled and compared. Bivariate multilevel linear regressions were used to evaluate the relationships between the continuity score and patient, provider or practice variables, including the practice model. These regressions were then stratified by model type to evaluate the transferability of the results between models.

#### Question one

Which factors are independently associated with continuity? Factors associated with continuity were identified by a multilevel random-intercept model. A model building approach was used. Patient characteristics were included first, then provider characteristics, and finally practice characteristics. Regressions were specified as two-level random-intercept models with individuals nested inside of practices. Within each regression, the model dummies were forced in and forward selection was performed for non-model variables. The regression equation was stratified by model to evaluate the transferability of findings across models. Effect sizes are presented as multilevel regression coefficients.

#### Question two

Does continuity of care differ by primary care model? Continuity scores were compared across models using multilevel regressions. Models were identified by a multilevel random-intercept model with entry and exit criteria of p = 0.05 and p = 0.10, respectively.

## Results

### Descriptive and bivariate analyses

One hundred and thirty-seven practices and 363 health practitioners were involved in the study. The sample comprised 35 FFS, 35 FHN, 35 CHC and 32 HSO practices. The overall patient response rate was 82% (range 77% - 94%). In total, 5361 patients completed surveys; 5296 of these had continuity scores. Comparison to provincial health administrative databases showed that the physicians participating in the study and their patterns of practice were similar to all Ontario physicians practicing in these models [33].

Table 1 shows the patient characteristics across models and their bivariate association with continuityl; this is reported as effect size (multilevel regression coefficient) and confidence intervals. Standardized effect sizes (ES/SD) are also provided in tables [36]. Table 2 shows the practice characteristics across models and their association with the continuity score. The results of the analyses stratified by models (results not shown) shows that all patient factor associations are consistent across models. Several practice factors had a significant bivariate association with the continuity score; fewer providers, 24 hours or more on call hours, and not being open on weekends were associated with higher continuity scores.

**Table 1:** Patient characteristics across models and bivariate associations with continuity score presented as multilevel regression coefficients representing effect size

**Patient characteristic**	**Profile distribution**				**Association with overall continuity score across all models**		**Standardized effect size**
	**CHC**	**FFS**	**FHN**	**HSO**	**Effect size (%)**	**95% CI **^**1**^	**ES/SD**^**2**^
Patient with continuity score (n)	1194	1366	1479	1257			
Patient profile (n)	1219	1375	1494	1273			
Age (years)†^3^	47	50	51	51	0.14	(0.12, 0.16)***	S
Sex (% female)†	73	67	66	61	0.44	(−0.29, 1.2)	S
At least one chronic disease (%)	66.8	65.3	68.7	65.2	4	(3.2, 4.7)***	S
Low Income (%)^4^†	33.6	12.5	11.6	11.3	1.4	(0.03, 2.4)*	S
Worked in the past 12 months (%)†	53.7	66.4	63.5	66.4	−3.9	(−4.7, -3.2)***	S
Patient of the practice less than 2 years (%)†	28.4	18.4	14.2	6.6	−3.5	(−4.5, -2.5)***	S
Mental Problems (%)§	47.3	44.8	42.8	40.7	−1.7	(−2.5, -1.0)***	S
Education (At least high school completed) (%)†	77.5	85.4	84.2	83.7	−3.4	(−4.4, -2.5)***	S

^1^ Symbols adjacent to the confidence interval indicate that the effect size is significantly associated with the continuity score:

* p < 0.05.

** p < 0.01. *** p < .001.

These are generated by multi-level linear regression.

^2^ ES/SD: Effect Size / Standard deviation; S if ES/SD < 0.2; M if 0.2 < =ES/SD < 0.5; L if 0.5 < =ES/SD < 0.8; VL if ES/SD > =0.8; CI = Confidence Interval.

^3^ Symbols adjacent to the patient characteristic indicate that it is statistically different across the models:

§ p < 0.05.

^‡^ p < 0.01.

^†^ p < 0.001.

These are generated by Chi-square statistic or by F-statistic (ANOVA), as appropriate.

^4^Income less than Low Income Cut Off [37].

**Table 2:** Practice and family profile of models and bivariate association with the continuity score

**Practice characteristics**	**Profile distribution**				**Association with continuity across all models**		**Standardized effect size**
	**CHC**	**FFS**	**FHN**	**HSO**	**Effect Size (%)**^**1**^	**95% CI**^**3**^	**ES/SD**
Practice type (n)	35	35	35	32			
Mean # FP and NP	5.5	2.5	3.9	1.8	−0.94	(−1.2, -0.55) **	M
Panel size^1^ (1000’s)†^2,4^	1.3	1.8	1.5	2	−0.33	(−0.12, 0.65)	S
Booking intervals (min)†	25	13	14	14	−0.17	(−0.31, -0.038) *	M
Solo practice^5^‡	0	26	37	38	3.7	(5.7, 1.7) **	VL
Presence of nurse(s) (%)†	97	31	69	75	−2.7	(−4.6, -0.81) **	M
Number of nurses^6^†	2.7	0.6	2	1.1	−1.1	(−1.6, -0.50) **	VL
# hours practice open out of (8am-4pm)	12	8	7	5	−0.32	(−0.48, -0.16) **	L
Open on weekends	20	20	23	0	−5.3	(−7.6, -3.0) **	M
Contextual factors							
Hospital (<10km)	71.4	85.3	94.1	86.7	−0.74	(−3.1, 1.6)	T
Rurality index^4^	14	12.6	16.2	8	−0.0085	(−0.063, 0.046)	T
Family physician characteristics	108	58	80	42			
Average years since graduation†	19	22	23	29	0.19	(0.082, 0.30) **	T
Female FP (%)^5^‡	58	45	41	26	−3	(−5.2, -0.86) **	L
Provider foreign trained (%)§	9.3	17.2	2.5	14.3	2.2	(−2.0, 6.4)	T
Provider has CFPC degree (%)	79	85	78	68	−0.77	(−3.3, 1.7)	T
>24 hours on call/week (%)	17.6	12.1	19	36.6	3.2	(0.82, 0.55) **	VL

^1^ ES/SD: Effect Size / Standard deviation; S if ES/SD < 0.2; M if 0.2 < =ES/SD < 0.5; L if 0.5 < =ES/SD < 0.8; VL if ES/SD > =0.8; CI = Confidence Interval; FP = family physician; NP = Nurse Practitioner; CFPC = College of Family Physicians of Canada.

^2^ Symbols adjacent to the practice factor indicate that it is statistically different across the models: ^§^ p < 0.05, ^‡^ p < 0.01, ^†^ p < 0.001. These are generated by Chi-square statistic or by F-statistic (ANOVA), as appropriate.

^3^ Symbols adjacent to the confidence intervals indicate that the effect size is significantly associated with the continuity score: * p < 0.05, ** p < 0.01. These are generated by multi-level linear regression.

^4^ Average number of patients per full time equivalent family physician in the practice.

^5^ Reflects practices that host a single family physician only.

^6^Number of full time equivalent nurses and nursing assistants.

^4^ Refers to the Rurality Index of Ontario adopted by the Ontario Medical Association (https://www.oma.org/economics/data/RuralityRIO.pdf).

^5^ We only captured the socio-demographic characteristics of respondents. The percent of a female family physician in the practice reflects the percentage of female family physician respondents.

### Question one. Which factors are independently associated with continuity?

Results of multilevel modeling are shown in Table 3. The first column shows the effect of patient and provider characteristics. The second shows the effect of practice factors in addition to patient and provider characteristics. The overall R-squared was 0.13 for the multi-level model with patient, provider and practice variables. In this model, several patient factors were significantly associated with higher continuity. Older patients reported greater continuity than younger patients (effect size = .052%). This means that for every year increase in age, patient continuity scores increased by 0.052 percent. Patients with more chronic diseases reported greater continuity (effect size = .70%). In contrast, those who spent more days per month in poor mental health reported lower continuity (effect size = −0.089%). People who worked full time (effect size = −2.15%) and who had a high school or greater education (effect size = −2.06%) reported lower continuity. Similarly, those people who had no regular provider^a^ (−13.48%) and those who had been a patient at the practice less than 2 years (effect size = −2.96%) reported lower continuity. Finally, continuity decreased as rurality increased (effect size = −0.028%).

**Table 3:** Factors significant and independently associated with the overall continuity score: multilevel modeling

	**% Change in continuity score**			
	**(multilevel regression coefficient: effect size)**			
	**Model A: patient and provider factors**		**Model B: patient, provider and practice factors**	
	**ES**	**ES/SD**^**1**^	**ES**	**ES/SD**
Variance explained (R^2^)	0.11		0.13	
N in model	5295		5295	
Model dummy variables				
CHC	−2.6	S	NS	
FFS	−1.4	S	NS	
FHN	−2.4	M	NS	
HSO (ref)	Base		Base	
Patient characteristics				
Age	0.055	S	0.052	S
Patient had paid work in the past 12 months (Full)	−1.97	S	−2.15	S
Practice patient < 2 years (1 if yes)	−2.97	S	−2.96	S
No regular provider	−1.45	S	−13.48	S
# of days having mental problems	−0.091	S	−0.089	S
# of chronic diseases	0.7108	S	0.7	S
Education binary (1 for high school or more)	−2.21	S	−2.06	S
Rurality index			−0.028	S
Provider profile				
Years since graduation	0.086	S	0.067	S
Practice profile				
Number of MDs	NS		−0.33	S
Total # of nurses (RPN,RN,NA)^2^	NS		−0.64	M
≤ 24 hours on-call/week	NS		−2.03	M
Practice open on weekends	NS		−2.39	M

^1^ES/SD: Effect Size / Standard deviation; S if ES/SD < 0.2; M if 0.2 < =ES/SD < 0.5; L if 0.5 < =ES/SD < 0.8; VL if ES/SD > =0.8; NS = Not Significant.

^2^*RPN* registered practical nurse, *RN* registered nurse, *NA* nurse assistant.

Physician age/experience was the only provider characteristic associated with continuity; we found that continuity increased with the number of years since physician graduation (effect size = 0.67%). Four practice factors were independently associated with the continuity score: number of providers, number of nurses, 24 hours or more of average on-call hours for the providers in the practice, and not being open on weekends. The continuity score decreased with the increase in the number of providers (effect size = −0.33%); it also decreased with the number of nurses (effect size = −0.64%). In addition, practices that were open on weekends had lower continuity scores (effect size = −2.39%). Finally, practices that had less than 24 on-call hours per week had lower continuity scores (effect size = −2.03%). The effect of these practice factors was largely consistent across models.

### Question two. Does continuity of care differ by primary care model?

Analyses that compared continuity across models showed that, in general, HSOs had better continuity than all of the other models, even when adjusting for patient demographics. However, when controlling for provider information, the difference between HSO and FFS was no longer significant (see Table 4).

**Table 4:** Differences in the continuity score across models in multilevel regressions

	**Beta estimates**^**1 **^**(95% CI)**			
Subject	CHC	FFS	FHN	HSO
Unadjusted	−4.8 (−7.3, -2.3)	−3.0 (−5.5, -0.5)	−3.1 (−5.6, -0.6)	Ref
Unadjusted (ES/SD)^2^	L	M	M	
Adjusted for patient demographics^3^	−4.3 (−6.7, -1.9)	−2.5 (−4.9, -0.2)	−2.9 (−5.3, -0.6)	Ref
Adjusted for patient demographics (ES/SD)	L	M	M	
Adjusted for patient demographics and practice factors^4^	−2.6 (−5.0, -0.2)	−1.4 (−3.8, 1)	−2.9 (−4.7, -0.1)	Ref
Adjusted for patient demographics and practice factors (ES/SD)	M	S	M	

^1^ Beta estimates are derived from multi-level regressions.

^2^ES/SD: Effect Size / Standard deviation; S if ES/SD < 0.2; M if 0.2 < =ES/SD < 0.5; L if 0.5 < =ES/SD < 0.8; VL if ES/SD > =0.8.

^3^ Factor considered were: Patient’s age, gender, work status, being a patient of the practice less than 2 years, having mental problems at least one day in the previous 30 days, having at least one chronic disease, and education.

^4^Factors were the same as above. There were no significant provider factors.

## Discussion

In this study, we sought to understand which patient, provider, and practice factors were associated with increased relational continuity. We also sought to compare relational continuity among different models of primary care in Ontario. This study had a number of strengths including a large sample size, the fact that it was representative of the different primary care models and the low patient refusal rate. Finally, the use of multi-level modeling allowed us to control for clustering within practices and to more clearly separate patient, provider and practice factors.

A number of patient characteristics predicted continuity, including age. Older patients reported higher continuity of care. This finding is consistent with findings in the UK [38] and New Zealand [39]. Guthrie [38] suggested that this might be due to greater health needs. It may also be due to the fact that patients can choose between rapid access and seeing their regular doctor [40]; older patients may have more ability (time, experience) to achieve this choice. In the present study, a higher number of chronic conditions was also associated with more continuity. The NZ study supports these findings [39]. It is likely that patients with greater needs see their providers more often, and thus develop a closer relationship with them. Because of their needs, they may place a particularly high value on continuity and trust and may thus wait to see their own provider. In contrast, patients with poorer mental health reported lower continuity. This may be due to real differences in continuity of care for people with poor mental health. It may also be due to higher patient needs/expectations about doctor-patient relationships among people with poor mental health [41]. Poor mental health, especially depression, is related to higher patient dissatisfaction [42,43]. Patients who had higher education and who worked full time reported lower continuity; this is similar to findings from New Zealand [39]. It is probable that those who work full time are less able to be flexible in the hours that they can visit their physician, and thus more likely to visit whichever providers are available. Another important finding is that patients who visited practices in more rural areas reported lower continuity. This may relate to the fact that in Canada, those who live in rural areas are underserved by physicians. For example, in 2004, only 16% of family physicians worked in rural areas while 21% of Canadians lived there. Moreover, the average distance to a physician was 10.4 km in weak Metropolitan Influenced Zone (more rural) communities [44]. Therefore, when people in rural areas are ill, they may be unable to see their regular family physician and may have to go to whoever is on call. Finally, and unsurprisingly, patients without a family physician and who had been with the practice for less than two years reported lower continuity.

Of the provider characteristics studied, only one was significantly related to continuity: years since graduation. Relational continuity was greater for each year since graduation. The finding that relational continuity increased as years since graduation increased is congruent with findings from the U.K. that showed that older physicians had greater continuity [38]. This makes sense as older physicians would have had more time to develop long-standing relationships with their patients. They are also more likely than younger physicians to have ‘office-only’ practices [45]. Furthermore, younger physicians are more likely than before to do locums; continuity of care is difficult to achieve as a locum practitioner [46].

Practice characteristics related to higher continuity of care included having more than 24 hours on-call for physicians, being closed on weekends, having a smaller practice, and having fewer nurses. The finding that practices which averaged more than 24 hours on-call per week had higher relational continuity is not surprising. In those practices which had more after-hours coverage, patients would be more able to see someone from their own practice rather than going to another clinic or to the emergency room. This finding is similar to that of Christakis, who found that, in pediatric practices, families whose providers worked 5 days a week compared to those whose providers worked ½ day a week experienced significantly higher continuity [47]. The finding that practices that were closed on weekends had greater continuity is likely due to the fact that patients who are ill on weekends will visit their own practice if open, regardless of whom they may see. It is possible that physicians in a group practice share weekend duty. This would also mean that a patient would get whoever was working that weekend and not necessarily their own physician.

The finding that smaller practices had higher continuity is consistent with previous work by Guthrie [38]. In larger practices, physicians have more colleagues on whom to rely to cover their patients, likely resulting in greater “sharing” of clinical duties. Finally, we found that having more nurses in a practice was related to lower continuity, irrespective of practice size. It may be that in practices with more nurses, physicians rely on them to a greater extent to cover routine aspects of care. This may increase the efficiency with which a practice functions but may also decrease relational continuity between the patient and physician.

We found that continuity of care was significantly higher in HSOs. This may be because, in Ontario, HSO members have been subjected to a financial penalty when their patient sought care with another practice. We suggest that this financial deterrent enticed practices to ensure greater accessibility and better continuity. Haggerty and colleagues demonstrated that practice organization that supports accessibility also supports continuity [48]. The fact that differences between models disapeared when physician characteristics were added to the model may be due to different physician profiles across models.

This study has a few limitations which need to be kept in mind when interpreting the results. The main limitation is the cross-sectional study design, which prevents us from establishing whether associations are causal. The determination of causality could be supported through a longitudinal study design or a randomized clinical trial. However, such designs are costly and may not be feasible for addressing the questions at hand, e.g., a randomized controlled trial assigning patients to practices with more or fewer nurses might support causal relationship, but would be impractical in the Ontario health care system where patients are free to choose their provider.

The self-reported measure of continuity we used might also be considered a limitation. However, we would argue that in the current climate of patient-centered care, the patient’s perception of continuity is vital.

## Conclusions

Research has consistently shown that continuity is important to patient and physician satisfaction and to patient outcomes. This study provides important information for physicians and policy makers who want to improve this important aspect of quality of care. Our finding that patients with greater physical health needs reported higher continuity is encouraging. However, the fact that patients with mental health issues reported lower continuity is concerning and should be explored further in order that these vulnerable patients receive equitable treatment. Furthermore, the lower continuity of care reported by patients in rural areas highlights concerns over access and quality of health care in rural areas of Canada. This, along with the finding that people without a regular primary care provider experienced lower continuity of care points to the need for more primary care practitioners in Ontario. In 2012, more than 927, 000 Ontario residents did not have a family doctor [49]. Finally, our finding that smaller practices had higher continuity suggests that physicians and policy makers need to consider the fact that ‘bigger is not always necessarily better’.

## Endnotes

^a^There are many reasons why a patient might not have a primary care provider. These include the ongoing shortage of primary care physicians in Ontario and the fact that some people don’t feel that they need a regular provider.

## Abbreviations

CHC: Community health centre; COMP-PC: Comparison of Models of Primary Care in Ontario; ER: Emergency room; FFS: Fee-for-service; FHN: Family health network; HSO: Health service organization; PCAT: Primary care assessment tool.

## Competing interests

The authors declare that they have no competing interests.

## Authors’ contributions

EK helped design and implement the study and analyses and led the writing of the paper. WH led the Comparison of Models study, including the design and implementation. He was also involved in directing analyses and in editing the article. SD directed the Comparison of Models Study and thus was involved in all aspects of design and implementation. She was also involved in directing the analyses and in editing the article. MT helped to design the analyses and ran all analyses. She also edited the article. LM-B helped to draft the introduction, worked on references and edited the paper. GG helped in adapting the PCAT for the COMPC study and in activities related to variable coding and data cleaning in the COMPC project, as well as in drafting the introduction of this paper. He also edited the paper. All authors read and approved the final manuscript.

## Pre-publication history

The pre-publication history for this paper can be accessed here:

http://www.biomedcentral.com/1471-2296/14/72/prepub
